# A rare case of para-vesical internal hernia presenting with complete small bowel obstruction managed in a resource-limited setting

**DOI:** 10.1093/jscr/rjaf599

**Published:** 2025-08-20

**Authors:** Dereje G Andargie, Chernet T Mengistie, Biruk T Mengistie, Yonas T Habtemariam, Worku M Sefefe, Biniam E Zelelew

**Affiliations:** Department of Surgery, College of Medicine and Health Sciences, University of Rwanda, KN 3 Rd, Nyarugenge District, Kigali City, Kigali Province 4285, Rwanda; School of Medicine, College of Health Sciences, Addis Ababa University, Tikur Anbessa Street, Arada Sub-City, Addis Ababa 9086, Ethiopia; School of Medicine, College of Health Sciences, Addis Ababa University, Tikur Anbessa Street, Arada Sub-City, Addis Ababa 9086, Ethiopia; Department of Surgery, College of Medicine and Health Sciences, Bahir Dar University, Pawi Road, Kebele 14, Bahir Dar City, Amhara Region 79, Ethiopia; Department of Surgery, College of Medicine and Health Sciences, Debre Markos University, Amanuel Street, Debre Markos Town, East Gojjam Zone, Amhara Region 269, Ethiopia; University of Global Health Equity (UGHE), Butaro Campus, Butaro Sector, Burera District, Northern Province 7078, Rwanda

**Keywords:** para-vesical internal hernia, small bowel obstruction, Cesarean section complications

## Abstract

Para-vesical internal hernias are rare but serious complications of abdominal surgeries and can present as small bowel obstruction (SBO). We report the case of a 30-year-old woman with a history of Cesarean delivery 9 years prior, who presented with crampy abdominal pain, bilious vomiting, and obstipation. Imaging revealed findings consistent with SBO. Exploratory laparotomy confirmed a para-vesical internal hernia with patchy ischemia of the bowel. The hernia was reduced, and the defect was repaired. The patient recovered uneventfully and was discharged on postoperative day five. This case highlights the importance of considering internal hernias in the differential diagnosis of SBO in patients with prior abdominal surgery. Early surgical intervention is critical to prevent bowel ischemia and further complications.

## Introduction

Small bowel obstruction (SBO) is a frequent surgical emergency, often caused by adhesions, hernias, or malignancies. Among the less common causes of SBO are internal hernias, which occur when a segment of the intestine protrudes through a defect in the peritoneum or mesentery. Internal hernias are particularly challenging to diagnose due to their nonspecific clinical presentation and the limitations of imaging studies in providing definitive preoperative diagnoses [1]. Para-vesical hernias, a rare subtype of internal hernias, occur when a viscus herniates between the median and medial umbilical ligaments. They are often associated with prior abdominal surgeries, including Cesarean deliveries, and can cause bowel ischemia or perforation if not promptly managed [2].

Internal hernias may develop through various anatomic defects, such as the broad ligament, which can occur following gynecological surgeries or Cesarean sections. Herniation through defects in the broad ligament, for instance, has been documented as a rare but significant cause of SBO, particularly in patients with a history of pelvic surgery [3]. Similarly, para-vesical hernias have been reported as a late complication of inguinal hernia repair, highlighting the diverse etiologies of internal hernias and the importance of considering them in the differential diagnosis of SBO, even in patients without prior abdominal scarring [4].

Diagnosing internal hernias preoperatively remains a challenge. While computed tomography (CT) scans can provide suggestive findings, they are often nonspecific, and the definitive diagnosis is frequently made intraoperatively [5]. This diagnostic uncertainty underscores the need for a high index of suspicion, particularly in patients with a history of abdominal or pelvic surgery who present with symptoms of bowel obstruction [6]. Early surgical intervention is crucial to prevent complications such as bowel strangulation, ischemia, or necrosis, which can significantly increase morbidity and mortality [7].

This case report highlights a para-vesical internal hernia presenting as a late complication of Cesarean delivery, emphasizing the importance of early diagnosis and prompt surgical management to avoid severe complications. It underscores the need to consider internal hernias in patients with a history of abdominal surgery who present with SBO symptoms, even in the absence of definitive imaging findings.

## Case presentation

A 30-year-old Sub-Saharan black, multiparous woman presented with a 15-hour history of crampy, intermittent abdominal pain, bilious vomiting, and obstipation. She had undergone a Cesarean delivery 9 years earlier for a persistent non-reassuring fetal heart rate; her recovery had been uneventful. She had no history of other surgeries, chronic medical conditions, or trauma, and she denied weight loss, rectal bleeding, smoking, or alcohol intake.

On admission, her vital signs were stable: blood pressure 110/70 mmHg, heart rate 74 bpm, and temperature 37.2°C. Abdominal examination revealed mild distension with diffuse tympanic percussion. A transverse Pfannenstiel scar was noted. The abdomen was soft, non-tender, and moved with respiration, and no hernias or masses were palpable. Bowel sounds were hyperactive, and a digital rectal examination showed an empty rectal vault.

**Figure 1:**
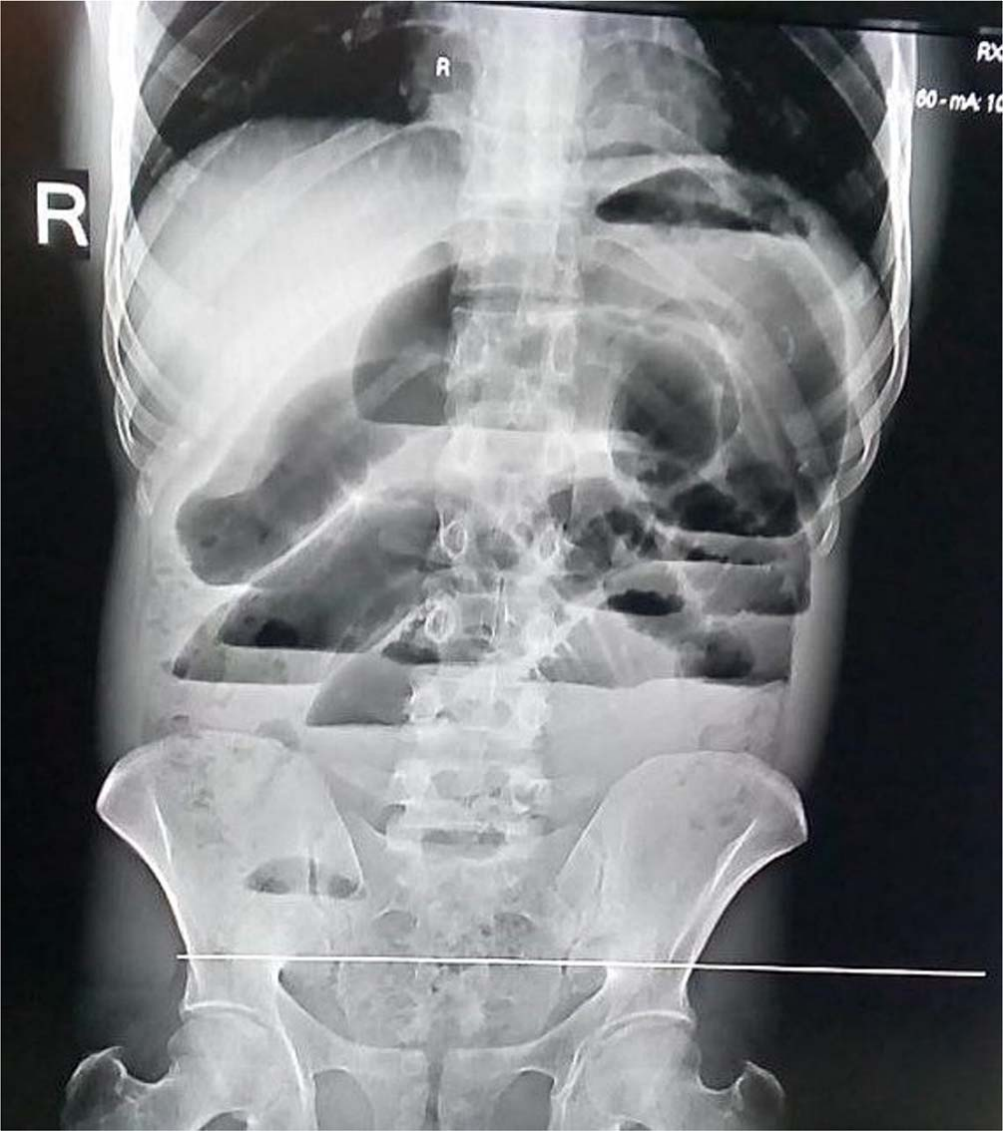
Abdominal X-ray—multiple air fluid levels.

Laboratory investigations, including complete blood count and serum chemistry, were within normal limits. An abdominal X-ray showed markedly dilated loops of small bowel with multiple air-fluid levels (Fig. 1), confirming SBO. A CT scan of the abdomen and pelvis was planned for further evaluation, but could not be obtained due to unavailability. The patient was resuscitated with intravenous fluids, nasogastric decompression, and broad-spectrum antibiotics and was taken emergently for surgery.

**Figure 2:**
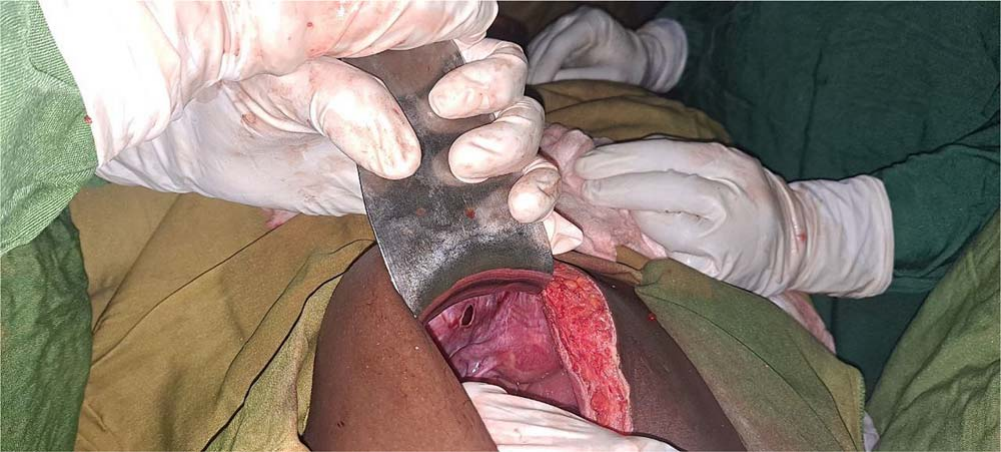
Intraoperative picture showing para-vesical defect.

**Figure 3:**
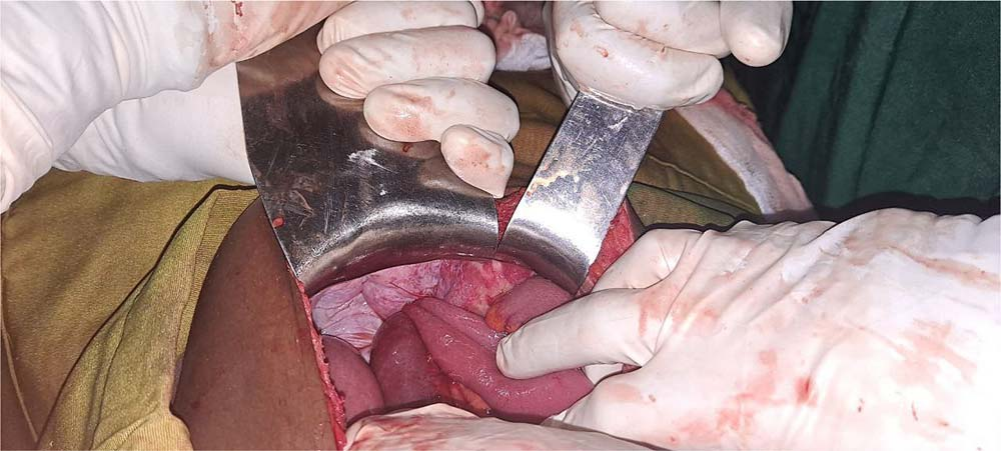
Intraoperative picture showing loop of incarcerated terminal ileum within the defect.

**Figure 4:**
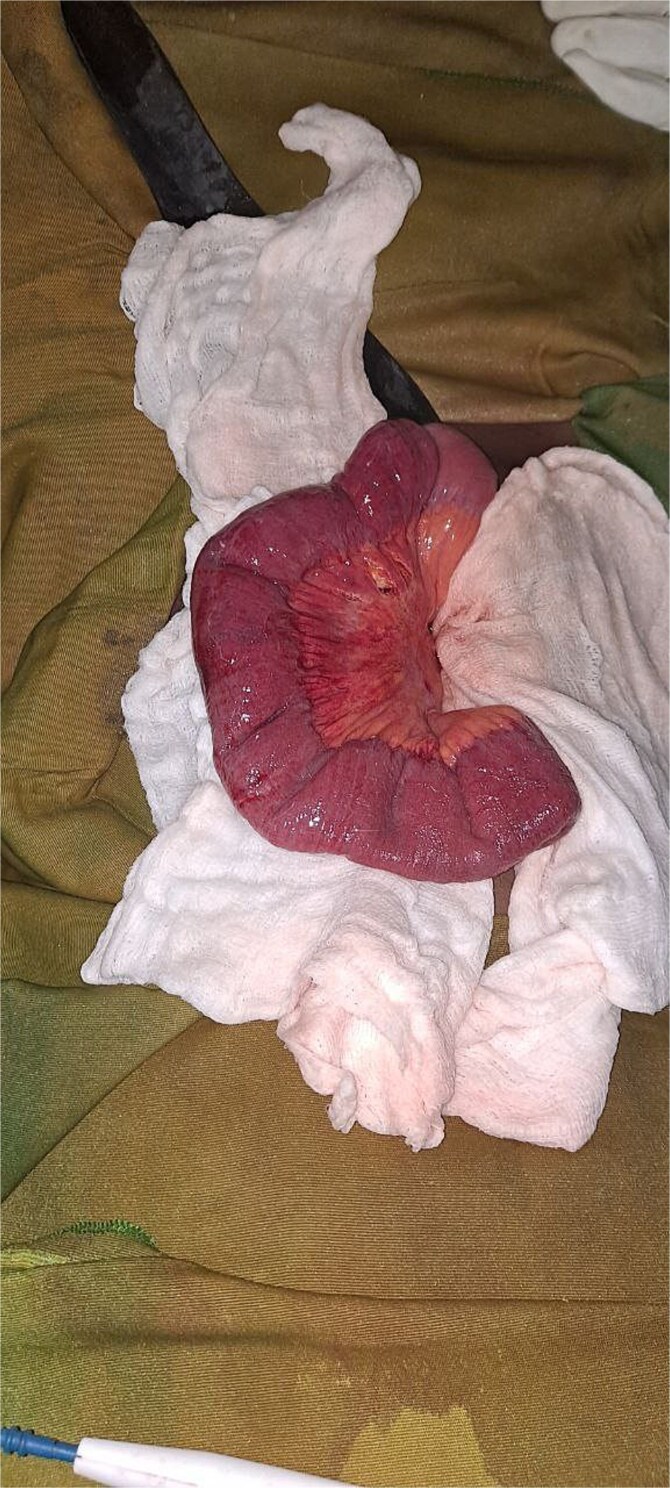
Intraoperative picture showing the herniated bowel after returning.

During exploratory laparotomy, ⁓200 mL of reactive peritoneal fluid was found. The small intestine was herniated through a 4 × 3 cm peritoneal defect on the left lateral side of the uterus (Fig. 2). A 10 cm segment of ileum (⁓100 cm proximal to the ileocecal valve) was trapped in the defect and showed patchy ischemia (Fig. 3). The herniated bowel was gently reduced back into the abdomen. Normal saline packs were applied until normal color and peristalsis returned (Fig. 4). The peritoneal defect was then repaired, and the abdomen was irrigated.

The patient had an uneventful postoperative recovery and was discharged on the fifth postoperative day with instructions to follow up in the surgical clinic.

## Discussion

Internal hernias are a rare but serious complication of abdominal surgeries. In particular, pelvic internal hernias include herniation through the para-vesical space, broad ligament, obturator canal, and other peritoneal defects. Para-vesical hernias after Cesarean delivery are especially uncommon and often present with nonspecific symptoms [1, 2, 4]. Internal hernias often occur when a segment of the intestine protrudes through a defect in the peritoneum or mesentery, leading to potential bowel obstruction, ischemia, or perforation [2, 5]. The formation of such defects can be attributed to prior surgical interventions, such as Cesarean sections, which may result in adhesions or peritoneal weaknesses that predispose patients to herniation [3].

The pathophysiology of internal hernias involves the protrusion of intestinal loops through congenital or acquired defects in the peritoneum or mesentery. In this case, the patient’s prior Cesarean section likely caused adhesions and a peritoneal defect, which allowed the small bowel to herniate into the para-vesical space. Such para-vesical hernias, in particular, occur when a viscus herniates between the median and medial umbilical ligaments, whereas broad ligament hernias involve a defect in the peritoneal leaf of the broad ligament, and obturator hernias protrude through the obturator canal. These are rare anatomical occurrences that can lead to bowel obstruction if not promptly addressed [2–4]. The risk of bowel ischemia and necrosis is high in such cases, as the herniated segment may become strangulated, compromising blood flow [5].

Diagnosing internal hernias preoperatively remains a significant challenge due to their nonspecific clinical presentation and the limitations of imaging studies. Differential diagnoses for SBO in post-Cesarean patients include adhesive obstruction, incisional hernia, Richter hernia, volvulus, and broad ligament hernia [1, 8]. While CT scans can provide suggestive findings, such as clustered bowel loops or mesenteric vessel abnormalities, they are often inconclusive, and the definitive diagnosis is frequently made intraoperatively [6]. In this case, the unavailability of a CT scan further complicated the diagnostic process, highlighting the importance of clinical judgment and exploratory laparotomy in resource-limited settings [7].

The management of para-vesical internal hernias involves prompt surgical intervention to relieve the obstruction and repair the defect. Early diagnosis and treatment are essential to prevent complications [8]. In this case, the patient underwent exploratory laparotomy, which confirmed the diagnosis and allowed for timely reduction of the herniated bowel and repair of the peritoneal defect. The prognosis for patients with timely surgical intervention is generally favorable, as demonstrated by the patient’s uneventful postoperative recovery and discharge on the fifth postoperative day [9].

This case is unique due to the late presentation of a para-vesical internal hernia 9 years after a Cesarean delivery. The absence of a CT scan due to unavailability highlights the importance of clinical judgment and the role of exploratory laparotomy in resource-limited settings. Additionally, the case underscores the need for clinicians to maintain a high index of suspicion for internal hernias in patients with a history of abdominal surgery [10], particularly in settings where advanced imaging modalities may not be readily available. Lastly, early surgical intervention is crucial to prevent severe complications and ensure favorable outcomes.

## Conclusion

This case highlights that internal hernias must be considered as a potential cause of SBO in patients with prior abdominal surgery, particularly Cesarean delivery. Para-vesical hernias, though rare, can present as late complications and require prompt surgical management. In our patient, the earlier Cesarean section likely produced adhesions and a peritoneal defect that permitted small bowel herniation. Early surgical intervention was crucial to prevent bowel ischemia and necrosis. Clinicians should maintain a high index of suspicion for internal hernias even if imaging is inconclusive; in settings without advanced imaging, exploratory laparotomy remains a critical diagnostic and therapeutic tool. This case underscores the need for awareness of internal hernias as a late post-Cesarean complication and the importance of timely surgery for favorable outcomes.

## Data Availability

The data underlying the results presented in this work are available within the manuscript.

## References

[1] Surel AA, Işık Nİ, Yazla M. Untangling diagnostic confusion in internal abdominal hernias. Ulus Travma Acil Cerrahi Derg 2023;29:1114–21. 10.14744/tjtes.2023.36037 37791450 PMC10644087

[2] Sardiwalla II, Phakula ML, Zimba MT, et al. Small bowel obstruction secondary to paravesical hernia. Int J Surg Case Rep 2016;26:156–8. 10.1016/j.ijscr.2016.07.021 27497038 PMC4976616

[3] Sajan A, Hakmi H, Griepp DW, et al. Herniation through defects in the broad ligament. JSLS 2021;25:e2020.00112. 10.4293/JSLS.2020.00112 PMC8241289 34248336

[4] Imamoglu M, Cay A, Sarihan H, et al. Paravesical abscess as an unusual late complication of inguinal hernia repair in children. J Urol 2004;171:1268–70. 10.1097/01.ju.0000113037.59758.6b 14767328

[5] Zhang Z, Hu G, Ye M, et al. A strangulated internal hernia beneath the left external iliac artery after radical hysterectomy with laparoscopic pelvic lymphadenectomy: a case report and literature review. BMC Surg 2021;21:273. 10.1186/s12893-021-01249-5 34059048 PMC8166092

[6] Gitonga EN, Shen H. Small bowel obstruction and strangulation secondary to an adhesive internal hernia post ESWL for right ureteral calculi: a case report and review of literature. BMC Gastroenterol 2021;21:176. 10.1186/s12876-021-01760-2 33865311 PMC8052854

[7] Torres-Villalobos GM, Kellogg TA, Leslie DB, et al. Small bowel obstruction and internal hernias during pregnancy after gastric bypass surgery. Obes Surg 2009;19:944–50. 10.1007/s11695-008-9681-x 18830790

[8] Park J, Chung M, Teixeira J, et al. Computed tomography findings of internal hernia after gastric bypass that may precede small bowel obstruction. Hernia 2016;20:471–7. 10.1007/s10029-015-1424-z 26659861

[9] LaMattina JC, Powell JM, Costa NA, et al. Surgical complications of laparoendoscopic single-site donor nephrectomy: a retrospective study. Transpl Int 2017;30:1132–9. 10.1111/tri.13005 28672056

[10] Augustin G, Matosevic P, Kekez T, et al. Abdominal hernias in pregnancy. J Obstet Gynaecol Res 2009;35:203–11. 10.1111/j.1447-0756.2008.00965.x 19335793

